# Genotyping of *Coxiella burnetii* from domestic ruminants and human in Hungary: indication of various genotypes

**DOI:** 10.1186/1746-6148-10-107

**Published:** 2014-05-07

**Authors:** Kinga M Sulyok, Zsuzsa Kreizinger, Heidie M Hornstra, Talima Pearson, Alexandra Szigeti, Ádám Dán, Eszter Balla, Paul S Keim, Miklós Gyuranecz

**Affiliations:** 1 Institute for Veterinary Medical Research, Centre for Agricultural Research, Hungarian Academy of Sciences, Budapest, Hungária körút 21 1143, Hungary; 2 Center for Microbial Genetics and Genomics, Northern Arizona University, 1298 S. Knoles Drive Flagstaff, AZ 86011-4073, USA; 3 Veterinary Diagnostic Directorate, National Food Chain Safety Office, Budapest, Tábornok utca 2 1143, Hungary; 4 National Center for Epidemiology, Budapest, Gyáli út 2-6 1097, Hungary

**Keywords:** *Coxiella burnetii*, Q fever, Genotyping, MLVA, MST, Hungary

## Abstract

**Background:**

Information about the genotypic characteristic of *Coxiella burnetii* from Hungary is lacking. The aim of this study is to describe the genetic diversity of *C. burnetii* in Hungary and compare genotypes with those found elsewhere. A total of 12 samples: (cattle, n = 6, sheep, n = 5 and human, n = 1) collected from across Hungary were studied by a 10-loci multispacer sequence typing (MST) and 6-loci multiple-locus variable-number of tandem repeat analysis (MLVA). Phylogenetic relationships among MST genotypes show how these Hungarian samples are related to others collected around the world.

**Results:**

Three MST genotypes were identified: sequence type (ST) 20 has also been identified in ruminants from other European countries and the USA, ST28 was previously identified in Kazakhstan, and the proposed ST37 is novel. All MST genotypes yielded different MLVA genotypes and three different MLVA genotypes were identified within ST20 samples alone. Two novel MLVA types 0-9-5-5-6-2 (AG) and 0-8-4-5-6-2 (AF) (Ms23-Ms24-Ms27-Ms28-Ms33-Ms34) were defined in the ovine materials correlated with ST28 and ST37. Samples from different parts of the phylogenetic tree were associated with different hosts, suggesting host-specific adaptations.

**Conclusions:**

Even with the limited number of samples analysed, this study revealed high genetic diversity among *C. burnetii* in Hungary. Understanding the background genetic diversity will be essential in identifying and controlling outbreaks.

## Background

The aetiological agent of Q (Query) fever is *Coxiella burnetii*, a Gram-negative, obligate intracellular, zoonotic bacterium which is widely distributed throughout the world. Domestic ruminants (cattle, sheep and goats) have the main role in the disease cycle, but *C. burnetii* has been isolated from other domestic and wild animals too [1]. Domestic ruminants are reservoirs of the agent with usually subclinical manifestation of the disease, but may suffer from reproductive disorders, and abortion and stillbirth can occur. The bacteria are transmitted mainly by inhalation. Additionally, infections may occur through the consumption of raw milk and milk products [2]. In humans, Q fever is typically an acute febrile illness with nonspecific clinical signs, such as atypical pneumonia and hepatitis in roughly 40% of the cases while the 60% of the individuals remain asymptomatic after infection [3]. A small percentage (~5%) of infected people may develop chronic infection with life-threatening valvular endocarditis [4,5].

Q fever is a notifiable disease in Hungary. Antibodies against *C. burnetii* were first detected in the sera of abattoir workers in 1950 [6], and infections were first diagnosed in 1956 in dairy and sheep farms [7]. According to recently published data, seroprevalence among cattle and sheep in Hungary were 38.0% and 6.0% with enzyme-linked immunosorbent assay respectively, which correspond with the European averages [8,9]. The number of yearly reported acute human infections in Hungary ranged between 36 and 68 during the past five years. Major outbreaks were last registered in the period of 1976–1980 and in 2013 in Hungary (unpublished data).

Genetic characterisation of *C. burnetii* is required for epidemiological investigations in Q fever outbreaks and for surveillance purposes. Several typing systems exist, including pulsed-field gel electrophoresis [10], sequence analysis or restriction fragment length polymorphism of single genes (16S ribosomal RNA, *icd*, *com1* or *muc*Z) [11-15]. The main problems with these methods are that their discriminatory power is poor and their reproducibility and transferability is not always straightforward [16]. Recently, two PCR-based typing methods have come into view: multispacer sequence typing (MST) [17] and multi-locus variable-number tandem repeat analysis (MLVA) [18,19]. Both methods have high discriminatory power and are reproducible, allowing interlaboratory comparisons without the need for isolation and cultivation of the organism under biosafety level three conditions.

Multiple sequence types have been identified from the Central European region in ticks and human samples: ST2 from Ukraine, ST16, ST18 from Romania and Slovakia, ST23 from Czech Republic and Slovakia and ST22, ST29 from Slovakia. While ST27 and ST32 were detected in goat samples in Austria [17,20]. MLVA types L, P and partial MLVA profiles were identified from cow milk samples originating from Slovakia, Austria and Croatia [21].

The aim of this study was to identify *C. burnetii* MST and MLVA genotypes occurring in Hungary and to compare them with types from foreign countries.

## Methods

**Figure 1:**
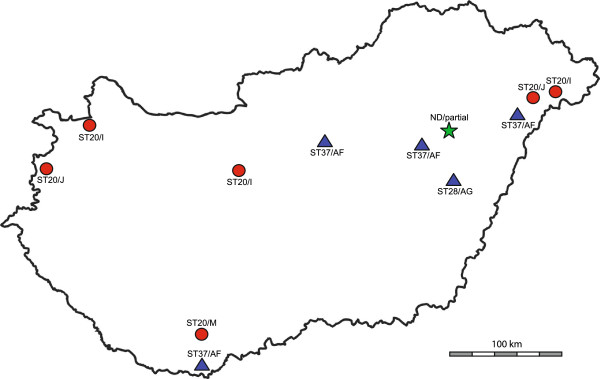
**Map of Hungary showing the geographical distribution of *C. burnetii *from cattle (red circles), sheep (blue triangles) and human (green star).** MST genotypes and MLVA types of samples are also presented. ND: not determined. (The blank map was downloaded from an open source [23]).

This study included five ovine and five cattle abortion samples (cotyledons) and a cattle milk sample collected from different parts of Hungary (Table 1, Figure 1). The blood sample of a 72 years-old man suffering from acute Q fever (1:128 IgGII titre with micro-immunofluorescence test, Focus Diagnostics, Cypress, CA) was also characterized. The samples were not related to any outbreak, they were collected through routine diagnostic examinations. All tests were performed in accordance with all applicable institutional and national guidelines and regulations, approved by the ethics committees of the Institute for Veterinary Medical Research (animal) and the National Center for Epidemiology (human) and with the consent of the patient.

**Table 1:** **MLVA and MST genotypes of *C. burnetii *specimens detected from cattle, sheep and a human clinical sample in Hungary**

**Sample ID**	**Source**	**Town of origin**	**Year**	**Ct**	**MLVA-6**						**MLVA type**	**MST type**
					**Ms23**	**Ms24**	**Ms27**	**Ms28**	**Ms33**	**Ms34**		
Coxi-14	cow milk	Martonvásár	2010	32.90	6	13	2	7	9	9	I	ST20
VS41	cow cotyledon	Nagyecsed	2012	27.78	6	13	2	7	9	9	I	ST20
VS76	cow cotyledon	Szombathely	2012	14.35	6	13	2	7	9	10	J	ST20
VS79	cow cotyledon	Nyírbátor	2012	30.34	6	13	2	7	9	10	J	ST20
VS93	cow cotyledon	Berkesd	2012	15.39	6	13	2	7	9	11	M	ST20
VS103	cow cotyledon	Kapuvár	2012	16.89	6	13	2	7	9	9	I	ST20
VS16	sheep cotyledon	Balkány	2012	8.85	0	8	4	5	6	2	AF	ST37
VS27	sheep cotyledon	Jászfelsőszentgyörgy	2012	29.83	0	8	4	5	6	2	AF	ST37
VS38	sheep cotyledon	Beremend	2012	12.79	0	8	4	5	6	2	AF	ST37
VS42	sheep cotyledon	Kunmadaras	2012	30.72	0	8	4	5	6	2	AF	ST37
VS108	sheep cotyledon	Biharnagybajom	2013	8.73	0	9	5	5	6	2	AG	ST28
4756	human blood	Hortobágy	2011	28.31	5	9	5	ND	6	2	partial	ND
*C.burnetii* RSA493*	tick	USA	1935		9	27	4	6	9	5		ST16

Ct: cycle threshold value of real-time PCR assay targeting the multicopy IS*1111* insertion element of the *C. burnetii* genome [22] to quantify the approximate bacterial load, 0: no amplification, ND: not determined probably because of insufficient DNA content, *: Genotype of RSA493 strain (Nine Mile, GenBank accession number: AE016828) was determined by *in silico* analysis.

Extraction of DNA from the samples was performed with the QIAamp Tissue and Blood kit (Qiagen GmbH, Hilden, Germany) according to the manufacturer’s instructions. To quantify the approximate bacterial load and to determine the cycle threshold (Ct) values, all samples were analysed by TaqMan based real-time polymerase chain reaction (PCR) assay targeting the multicopy IS*1111* insertion element of the *C. burnetii* genome [22].

MST consisted of amplification followed by sequencing of ten different spacer regions of the *C. burnetii* genome: Cox2, 5, 18, 20, 22, 37, 51, 56, 57 and 61 [17]. Primer sequences and reaction conditions were described earlier [17]. The compiled sequences of the PCR products were analysed with the BioEdit Sequence Alignment Editor 7.1.11 [24]. Sequence types (STs) were determined using the MST database and previous publications [17,20,25]. Unfortunately, the allele codes for ST35 and ST36 are not publicly available therefore we were not able to include them in the phylogenetic analysis [25]. To determine the phylogenetic relationships of our samples to known MST genotypes [17,20] we used the sequence information of our three STs and constructed a phylogenetic tree with the 112 polymorphisms and methods described in Hornstra et al. [26]. As samples VS16/27/38/42 (sheep abortion) appeared to have a new ST and homoplasies can alter topologies of trees, we also constructed a sub-tree to verify its placement using the same methods and 112 polymorphisms as above but only with STs: 8, 9, 10, 27, 28, 31, and the two sheep abortion STs from this study.

MLVA was performed by single PCRs targeting six variable microsatellite markers as performed in other recent studies [18,27-30]. Ms27, Ms28 and Ms34 contain repeat units of six base pairs and Ms23, Ms24 and Ms33 contain repeat units of seven base pairs. The 3′-end-labelled forward and reverse primer sequences, and PCR conditions were applied as described before [27-30]. The amplification products were run on an ABI 3100 Genetic Analyser and electropherograms were evaluated with the Peak Scanner Software 2.0 (Applied Biosystems Inc., Foster City, CA). DNA of the Nine Mile strain (RSA 493, Coxevac, Ceva Inc., Budapest, Hungary) was used as a reference. The repeat numbers of each marker were determined by extrapolation using the obtained length of the sample fragments relative to the obtained fragment-length of the reference strain. A modified coding convention was used for Ms33: nine imperfect repeat units were counted in the Nine Mile strain (G. Vergnaud, personal communication). Novel MLVA types were determined if the described allele combinations had not been previously described in any publications applying the same MLVA method [21,27-29].

## Results

All 12 samples were positive with the *Coxiella* specific real-time PCR with Ct values below 33 and all but one yielded complete MST and MLVA genotypes (Table 1). MST alignment and MLVA profiles were deposited in Dryad ([31], doi:10.5061/dryad.rc8q4) and in the *Coxiella* MLVA database ([32], accession numbers: Hung00-Hung11).

**Figure 2:**
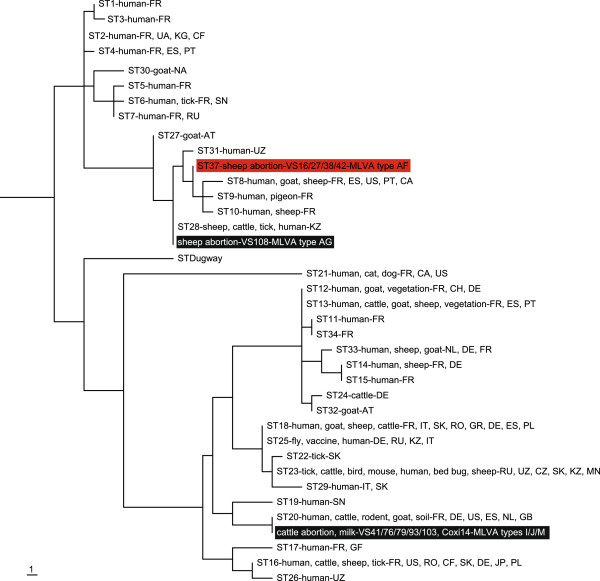
**Parsimony tree showing the placement and phylogenetic relationships of the sequence types (ST) from this study with known STs **[19]**, rooted according to Pearson et al. **[33]**.** Highlights indicate the placement of the STs of our samples; the red highlight denotes the newly proposed ST37. One hundred and twelve polymorphisms from Hornstra et al. [26] were used to construct this phylogeny and resulted in 156 equally parsimonious trees, the first of which is depicted above. Tree length is 120 and the homoplasy index (excluding uninformative characters) is 0.0769. Isolate origins and sources are given according to previous publications [19,20,25,26,28,29,35-38] using the following location codes: Austria (AT), Canada (CA), Central African Republic (CF), Czech Republic (CZ), France (FR), French Guiana (GF), Germany (DE), Greece (GR), Italy (IT), Japan (JP), Kazakhstan (KZ), Kyrgyzstan (KG), Mongolia (MN), Namibia (NA), Netherlands (NL), Poland (PL), Portugal (PT), Romania (RO), Russian Federation (RU), Senegal (SN), Slovakia (SK), Spain (ES), Switzerland (CH), Ukraine (UA), United Kingdom (GB), United States (US) and Uzbekistan (UZ).

The MST analysis of the six cattle and five ovine samples revealed a novel (proposed ST37) and two previously described (ST20, ST28) STs (Table 1, Figure 2). The allele codes of the newly identified genotype ST37 are 5-4-9-5-8-5-2-3-4-6 for spacers Cox2-Cox5-Cox18-Cox20-Cox22-Cox37-Cox51-Cox56-Cox57-Cox61, respectively. Phylogenetic analysis according to Honstra et al. [26] resulted in 156 equally parsimonious tree topologies, one of which is shown in Figure 2, with a homoplasy index (excluding uninformative characters) of 0.0769. Sub-tree construction using STs 8, 9, 10, 27, 28, 33, and the two sheep abortion STs from this study resulted in a single tree with no homoplasy whose topology exactly matched the topology for this clade in the tree selected in Figure 2. In clonal groups of organisms with little genetic diversity the homoplasy index has been shown to be the most appropriate measure of phylogenetic accuracy [33,34], therefore the results of this sub-tree provide confidence in the accuracy of the placement of this novel ST (proposed ST37) in Figure 2.

Five MLVA genotypes were identified among the 11 cattle and ovine samples with complete genotypes, out of which two novel MLVA types were described in this study. Three MLVA types (I, J, M) were detected among the six cattle samples, and the two novel genotypes (AF and AG) were defined from the five ovine samples. In the case of the human sample only a partial genotype was obtained despite the relatively high DNA load (Ct value was 28.31). The identified allele combination in the human sample did not match any previously defined genotypes, implying the discovery of a novel MLVA type as well. The partial MLVA genotype from the human sample showed high similarity to the novel genotype AG defined in sheep, as four out of the five microsatellites were the same.

All samples originating from cattle were identified as ST20, regardless of their MLVA types. Samples from sheep belonged to two MST types: ST28 was detected in a sample with AG MLVA genotype, and the proposed ST37 was described in samples with AF MLVA type. Host specificity of the genotypes was observed as all MLVA genotypes from ST20 were described from cattle only and the two other genotypes were detected in sheep exclusively.

## Discussion

Molecular characterisation of *C. burnetii* is a useful tool to explore the genotypic diversity in a region and to determine relationships between variants of this bacterium. The main advantage of both MST and MLVA typing methods is their high discriminatory power and that the cultivation of the bacteria is unnecessary for their application.

MST and MLVA genotyping methods revealed genotypic diversity among samples from domestic ruminants and human in Hungary. Three *C. burnetii* STs (ST20, ST28 and ST37) are present in Hungarian ruminants.

Indicating the broad range of ST20 occurrence, other samples have been reported from The Netherlands, Germany, Spain, in human clinical samples from France and in cow’s milk, soil, and goat placental material from the United States [17,26,29,35,39]. Microvariants of the ST20 genotype occur as well and have been found elsewhere; MLVA genotypes I, J and M were described before from cow milk and milk products from several countries in Europe (France, the Netherlands, Portugal, Spain and Switzerland) and other parts of the world (e.g. Qatar, Saudi Arabia) [21]. ST20 was also detected in an outbreak among goats recorded from the United Kingdom [36]. In conclusion, although ST20 is most often associated with cattle products, this genotype occasionally infects other species as well.

ST28 samples have been collected from cows, sheep, *Hyalomma* sp. tick and human blood in Kazakhstan [17]. ST37 genotype is closely related to ST27-28, 31 and 8–10 genotypes that have been collected from humans in Austria, Kazakhstan, Uzbekistan, France and Portugal as well as sheep and goats from Spain, France, Canada and the USA (Figure 2) [17,20,28,29,39]. The comparison of the novel genotypes (AG and AF) with others described in Europe revealed similarities with genotypes AA and T, differing at 2 and 3 loci respectively and also associated with sheep, goats and human [28,29]. Again, that these genotypes are most commonly associated with sheep and goats provides further evidence for host preference [39]. We speculate that there may be some association between the Hungarian human and ovine infections (MLVA genotype AG) given genotype similarities and as the geographical distance between the two samples was only 50 km.

No matches were found among the MST and MLVA profiles from Hungary and from the dominant genotype (ST33) associated with the Q fever outbreak of The Netherlands that emerged in 2007 [27,35]. STs described earlier from other Central European countries differed also from the STs found in Hungary [17,20].

## Conclusions

The present study provides information about the genotypic diversity of *C. burnetii* occurring in Hungary. From the examined 12 *C. burnetii* samples originating from cattle, sheep and human three MST types (containing one novel profile) and five MLVA types (including two novel profiles) have been identified. These genotypes cluster with host, suggesting host-specific evolutionary adaptations as shown by Pearson et al. [39] as well. The additional analyses of samples from a broader range of livestock species are needed to confirm this finding. Our results provide data for the European surveillance and help to increase information about Q fever in this Central European region.

## Abbreviations

MST: Multispacer sequence typing; MLVA: Multiple-locus variable number tandem repeat analysis.

## Competing interests

The authors declare that they have no competing interests.

## Authors’ contributions

KMS genotyped the samples, analysed the data and wrote the manuscript. ZK, AS, ÁD, EB collected the samples, performed the PCR screening and edited the manuscript. HMH, TRP and PSK helped in genotyping and in the writing of the manuscript. MG designed the study and wrote the manuscript. All authors read and approved the final manuscript.
